# Hydroxychloroquine-Induced AGEP with Positive Rechallenge: A Case Report and Mini Review of the Literature

**DOI:** 10.3390/dermatopathology13030030

**Published:** 2026-07-03

**Authors:** Kristijan Jovanović, Tamara Umeljic Sočević, Milos Stepovic, Jovana Milosavljević, Jovica Tomović, Miroslav M. Sovrlić, Marko Folić, Miloš N. Milosavljević, Dalibor Jovanović, Nevena Folić

**Affiliations:** 1 Department of Anatomy, Faculty of Medical Sciences, University of Kragujevac, 34000 Kragujevac, Serbia; kralj100@yahoo.com (K.J.); stepovicmilos@yahoo.com (M.S.); jowana.ilic@yahoo.com (J.M.); 2 Center of Dermatovenereology, University Clinical Center Kragujevac, 34000 Kragujevac, Serbia; 3 Department of Pharmacy, Faculty of Medical Sciences, University of Kragujevac, 34000 Kragujevac, Serbia; 4 Department of Clinical Pharmacology, University Clinical Center Kragujevac, 34000 Kragujevac, Serbia; markof@fmn.kg.ac.rs; 5 Center for Pharmaceutical and Pharmacological Research, Faculty of Medical Sciences, University of Kragujevac, 34000 Kragujevac, Serbia; 6 Department of Pharmacology and toxicology, Faculty of Medical Sciences, University of Kragujevac, 34000 Kragujevac, Serbia; milosavljevicmilos91@gmail.com; 7 Department of Pathology, Faculty of Medical Sciences, University of Kragujevac, 34000 Kragujevac, Serbia; 8 Department of Pediatrics, Faculty of Medical Sciences, University of Kragujevac, 34000 Kragujevac, Serbia; nevena.folic@yahoo.com; 9 Pediatric Clinic, University Clinical Center Kragujevac, 34000 Kragujevac, Serbia

**Keywords:** hydroxychloroquine, acute generalized exanthematous pustulosis, pustular dermatosis, positive rechallenge, adverse drug reaction

## Abstract

Background/Objectives: Hydroxychloroquine is widely used in the treatment of autoimmune and dermatologic diseases; however, it may rarely induce severe cutaneous adverse reactions. Acute Generalized Exanthematous Pustulosis is an uncommon, acute pustular eruption most frequently associated with antibiotics. Hydroxychloroquine-induced AGEP remains relatively rare and diagnostically challenging due to its atypical and prolonged clinical course. Case presentation: We report the case of a 45-year-old woman with rheumatoid arthritis and a complex medical history who developed generalized urticarial and pustular dermatosis following re-exposure to hydroxychloroquine. Notably, the patient had experienced a similar cutaneous reaction after previous exposure to the same medication several years earlier. Ten days after completing a treatment course of hydroxychloroquine, she developed rapidly progressive pruritic erythematous and urticarial plaques that evolved into generalized annular lesions with peripheral scaling and grouped sterile pustules. Laboratory evaluation demonstrated leukocytosis, intermittent eosinophilia, and elevated IgE levels, while the infectious workup was negative. Histopathological examination revealed subcorneal pustules with neutrophilic infiltration, mild spongiosis, and scattered individual eosinophils, perivascular inflammatory infiltrates, findings consistent with AGEP. Retrospective assessment using the EuroSCAR scoring system classified the reaction as probable AGEP, while the Naranjo adverse drug reaction scale supported a probable causal relationship with hydroxychloroquine. Clinical improvement was achieved after withdrawal of the drug and treatment with systemic corticosteroids and supportive therapy. Conclusions: This case highlights the importance of recognizing atypical presentations compatible with hydroxychloroquine-induced probable AGEP and emphasizes the diagnostic value of a positive rechallenge as supportive evidence of drug causality. Early recognition and prompt discontinuation of the offending agent are essential to prevent severe complications and recurrence.

## Simple Summary

Hydroxychloroquine is widely used in the treatment of autoimmune and rheumatologic diseases, but in rare cases, it may cause severe cutaneous adverse reactions. AGEP is an uncommon drug-induced pustular eruption most frequently associated with antibiotics. Hydroxychloroquine-induced AGEP often presents atypically and may follow a prolonged clinical course. This paper presents a rare case of hydroxychloroquine-induced probable AGEP with urticarial and pustular skin lesions in a patient with rheumatoid arthritis, with the diagnosis supported by a positive rechallenge after re-exposure to the drug. The report highlights the importance of clinical recognition, histopathological evaluation, and diagnostic scoring systems in supporting the diagnosis. This study summarizes recent literature on hydroxychloroquine-induced AGEP and emphasizes the importance of early drug discontinuation and careful assessment of previous adverse drug reactions to prevent recurrence and complications.

## 1. Introduction

Hydroxychloroquine is widely used in the treatment of rheumatologic and dermatologic diseases due to its immunomodulatory and anti-inflammatory properties. Its mechanisms of action include modulation of lysosomal activity, inhibition of antigen presentation, and interference with cytokine signaling pathways, which contribute to its anti-inflammatory effects [1]. Although generally considered safe, its use may be associated with various adverse effects, including involvement of the skin, eyes, cardiovascular system, and other organs [2,3]. Cutaneous adverse reactions to hydroxychloroquine range from mild eruptions to severe hypersensitivity syndromes, although serious reactions remain uncommon [3].

Acute Generalized Exanthematous Pustulosis (AGEP) is an acute, drug-induced dermatosis characterized by the sudden onset of numerous sterile, non-follicular pustules on an erythematous background, often accompanied by leukocytosis and fever [4]. AGEP is considered a severe cutaneous adverse reaction mediated by drug-specific T cells and cytokine release, particularly involving IL-8 and IL-36 pathways responsible for neutrophil recruitment [5,6].

Although antibiotics are the most common causes of AGEP, hydroxychloroquine is increasingly being recognized as a potential trigger of this reaction, particularly in patients with autoimmune diseases [4]. Hydroxychloroquine-induced AGEP has gained increasing attention in recent years due to its atypical presentation and prolonged clinical course compared with classical AGEP [7].

Relatively few cases of hydroxychloroquine-induced AGEP have been described in the literature, and the disease may present atypically, with a prolonged course or overlap with other dermatoses, such as pustular psoriasis [8]. This overlap may complicate the differential diagnosis, making histopathological evaluation and validated diagnostic scoring systems particularly important [9].

In addition, the presence of genetic factors, such as mutations in the IL36RN or CARD14 genes, may contribute to the severity of the clinical presentation [10]. These genetic alterations have been associated with dysregulated inflammatory signaling and more severe pustular phenotypes [11].

Of particular diagnostic importance is the phenomenon of a positive rechallenge, i.e., the recurrence of symptoms after re-exposure to the same drug, which strongly supports a causal relationship [12]. Because intentional rechallenge is rarely performed for ethical reasons, documented cases of positive rechallenge provide particularly strong evidence for drug causality [13].

The aim of this paper is to present a rare case of hydroxychloroquine-induced probable AGEP characterized by prominent urticarial and annular morphology, compatible histopathological findings, and recurrence following re-exposure, highlighting the diagnostic challenges in distinguishing AGEP from hydroxychloroquine-triggered pustular psoriasis.

## 2. Hydroxychloroquine-Induced AGEP: Mini-Review of the Literature

Literature search was performed in the PubMed database using the following keywords: acute generalized exanthematous pustulosis and hydroxychloroquine and case report. The search was conducted to identify published case reports of hydroxychloroquine-induced AGEP. A total of 14 articles were initially retrieved, of which 10 met the inclusion criteria and were included in the final analysis. Studies were included if they reported individual patient data on HCQ-associated AGEP with sufficient clinical, histopathological, and outcome information. This section is intended as a selected, illustrative overview of the published literature rather than a systematic or comprehensive review.

Hydroxychloroquine-induced acute generalized exanthematous pustulosis represents a rare but increasingly recognized severe cutaneous adverse reaction. Recent literature suggests that HCQ-induced AGEP may differ from classical AGEP caused by antibiotics, particularly regarding latency period, clinical course, and therapeutic response [14].

Several recent reports described predominantly female patients with underlying autoimmune or rheumatologic diseases who developed diffuse sterile pustular eruptions after initiation of hydroxychloroquine therapy. The interval between HCQ exposure and onset of skin manifestations was generally longer than that observed in classical AGEP, commonly ranging from 10 days to 3 weeks. In the study by Luo et al., which included five rheumatologic patients with HCQ-induced AGEP, the mean latency period was 12.2 days, while the mean time to complete resolution reached 25.2 days [14].

A broader range of case reports supports these observations. Bailey et al. described an early report of HCQ-induced AGEP with diffuse pustular eruption and fever, confirming rapid clinical improvement after drug withdrawal and corticosteroid therapy, highlighting the typical self-limited course once the drug is discontinued [15]. Similarly, Duman et al. reported a more complex clinical presentation with targeted lesions and neutrophilic leukocytosis, emphasizing that HCQ-induced AGEP may mimic erythema multiforme or pustular psoriasis and may require systemic corticosteroids and adjunctive therapies such as dapsone in persistent cases [16].

Atypical clinical presentations have also been increasingly reported. In addition to generalized pustules on an erythematous background, some patients demonstrated prolonged disease duration, resistance to corticosteroid therapy, overlap with pustular psoriasis, or recurrent pustular eruptions after re-exposure to HCQ. Chen et al. reported repetitive atypical pustular drug eruptions in the same patient occurring 12 years apart after hydroxychloroquine administration, emphasizing the diagnostic overlap between AGEP and pustular psoriasis.

More severe phenotypes have also been documented. Mohaghegh et al. reported an AGEP–SJS overlap induced by hydroxychloroquine with extensive skin sloughing and mucosal involvement requiring burn unit care and IVIG therapy, demonstrating that HCQ-induced AGEP may rarely progress into life-threatening overlap syndromes [7]. Likewise, Coleman et al. described another AGEP–SJS overlap case with extensive mucosal involvement and systemic complications, further supporting the concept that HCQ-related cutaneous reactions can exist on a spectrum ranging from classical AGEP to severe epidermal necrolysis-like disease [17].

Histopathological findings reported in recent cases were generally consistent with AGEP and included subcorneal or intraepidermal pustules, papillary dermal edema, and neutrophilic and eosinophilic infiltrates. However, differentiation from generalized pustular psoriasis may remain challenging, especially in patients with recurrent episodes or atypical morphology [18].

Recent studies additionally highlighted the potential role of genetic susceptibility in HCQ-induced AGEP. Luo et al. identified CARD14 mutations in several patients with HCQ-induced AGEP, suggesting that dysregulation of inflammatory signaling pathways may contribute to disease severity and atypical presentations [14]. Furthermore, pharmacogenomic associations involving HLA alleles have recently been described. Zheng et al. reported HCQ-induced AGEP associated with HLA-B58:01, HLA-C08:01, and HLA-A*02:06 genotypes, supporting the hypothesis that genetic predisposition may influence susceptibility to severe cutaneous adverse drug reactions [19].

Case reports by Hsieh et al. further support the consistent latency pattern of approximately 2–3 weeks and confirm that HCQ-induced AGEP typically presents with fever, neutrophilia, and rapid histopathological confirmation via subcorneal and intraepidermal pustules, reinforcing the diagnostic reliability of biopsy in ambiguous clinical cases [20]. More recently, Ali et al. described an atypical variant of HCQ-induced AGEP sine pustules, in which clinical pustules were absent but histopathology confirmed subcorneal pustulation with scattered individual eosinophils infiltrate, expanding the recognized clinical spectrum and emphasizing the importance of biopsy in morbilliform drug eruptions suspected to be AGEP [21].

An important characteristic of HCQ-induced AGEP is its frequently prolonged clinical course, likely associated with the long elimination half-life of hydroxychloroquine. Persistent disease despite drug discontinuation and systemic corticosteroid therapy has been described, and some refractory cases required additional immunosuppressive treatment, including methotrexate [18].

Positive rechallenge remains rarely documented because intentional re-exposure to the suspected drug is generally considered ethically unacceptable. Therefore, inadvertent rechallenge cases provide particularly strong evidence supporting causality between hydroxychloroquine exposure and AGEP development [22].

The present case contributes to the growing literature on HCQ-induced AGEP by demonstrating generalized pustular and urticarial manifestations with positive rechallenge, further emphasizing the importance of early recognition and prompt discontinuation of hydroxychloroquine in patients presenting with acute pustular eruptions (Table 1).

In contrast to the majority of published cases showing spontaneous resolution of hydroxychloroquine-induced AGEP following drug discontinuation, our patient exhibited a persistent and treatment-resistant disease course.

## 3. Case Presentation

We present the case of a 45-year-old female patient with a complex medical history including arterial hypertension, insulin resistance, and hypothyroidism (treated with Levothyroxine), who developed generalized cutaneous lesions following re-exposure to hydroxychloroquine. The patient had a significant allergic history, including childhood penicillin-induced anaphylaxis and hypersensitivity to inhalant allergens (dust mites, pollen, and tobacco smoke). She also reported a previous adverse reaction following hydroxychloroquine therapy in 2017, when a generalized erythematous eruption developed approximately 10 days after treatment initiation. Available medical records documented a nonspecific histopathological finding, and a definitive diagnosis could not be established at that time. Clinical improvement occurred after drug discontinuation and treatment. Although complete documentation from the initial episode was unavailable, the similar latency period, clinical presentation, and hydroxychloroquine exposure strongly suggest that both episodes represented the same adverse drug reaction; however, this cannot be confirmed with certainty because of the limited available records. In the current episode, hydroxychloroquine was reintroduced on 21 January 2026, at a dose of 250 mg daily for the treatment of rheumatoid arthritis, with close clinical awareness given the patient’s previous suspected adverse reaction. The patient received hydroxychloroquine for 10 days, which was discontinued due to the emergence of early cutaneous symptoms suggestive of a drug-induced hypersensitivity reaction. No immediate adverse effects were observed during the treatment period; however, on 31 January, shortly after discontinuation of therapy, she developed pruritic urticarial lesions, initially presenting as small erythematous plaques. Despite possible additional contributing factors, such as contact with nettle extracts, the rash rapidly progressed.

**Figure 1:**
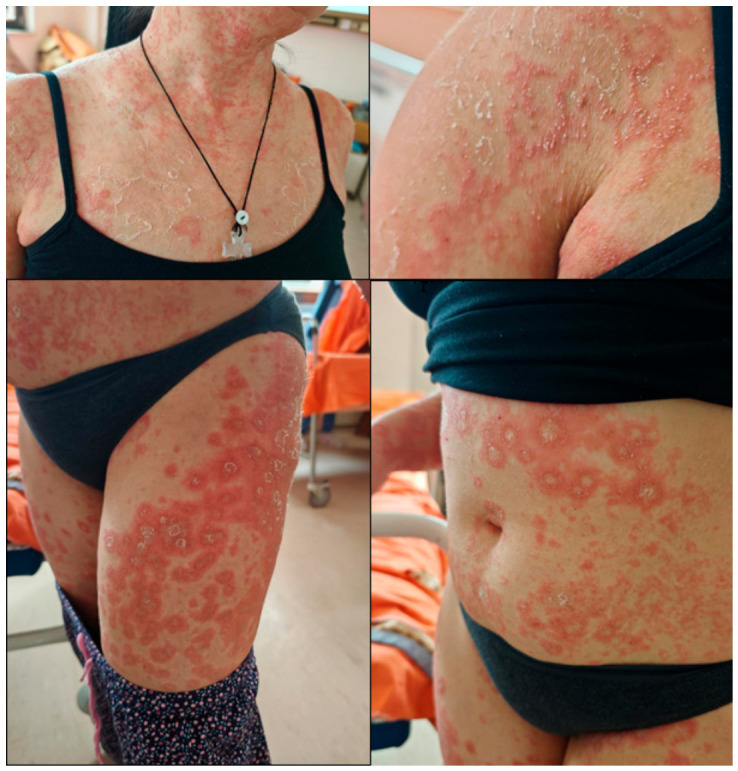
Panel presentation of the cutaneous lesions.

Due to worsening cutaneous manifestations, the patient was hospitalized from 6 February to 13 February 2026. On admission, dermatological examination revealed disseminated erythematous and urticarial lesions, predominantly involving the trunk and upper extremities, with progression to generalized annular erythematous plaques with peripheral scaling and grouped papulopustules. In some areas, the lesions coalesced into larger plaques with marked desquamation, particularly on the lower extremities (Figure 1). The condition was accompanied by severe pruritus.

Laboratory findings demonstrated mild anemia. Leukocytosis peaked at 13.6 × 10^9^/L, while peripheral eosinophilia was observed, reaching 0.2 × 10^9^/L during the active phase of the disease, with a maximum relative eosinophil count of 8.7%. Total IgE levels were elevated, and inflammatory markers were only mildly increased. The infectious workup did not indicate active infection. Radiological and other systemic evaluations showed no evidence of acute organ involvement.

A skin biopsy was obtained from an active pustular lesion on the affected skin.

Histopathological examination demonstrated subcorneal and intraepidermal pustule formation composed predominantly of neutrophils, accompanied by focal spongiosis and mild epidermal acanthosis. The pustules were localized mainly within the superficial epidermis and were associated with prominent neutrophilic exocytosis into the adjacent epidermal layers. Exocytosis of inflammatory cells into the epidermis was present, while focal keratinocyte damage was observed without evidence of full-thickness epidermal necrosis. Occasional apoptotic keratinocytes were identified; however, extensive interface alteration or confluent epidermal necrosis was absent.

**Figure 2:**
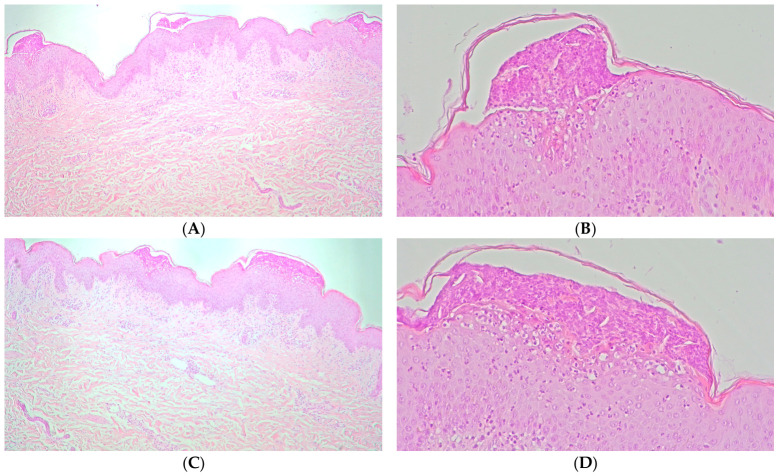
Histopathological findings consistent with acute generalized exanthematous pustulosis (AGEP). (**A**) Low-power view (×50) demonstrating papillary dermal edema and a superficial perivascular inflammatory infiltrate. (**B**) Subcorneal pustule formation composed predominantly of neutrophils (original magnification ×100). (**C**) Low-power view (×50) of a different histological section showing papillary dermal edema and a superficial perivascular inflammatory infiltrate adjacent to the epidermis. (**D**) Higher magnification (×100) showing neutrophilic pustules with focal keratinocyte damage. Hematoxylin and eosin staining.

The superficial dermis showed papillary edema and a mixed perivascular inflammatory infiltrate composed mainly of neutrophils, lymphocytes, and scattered eosinophils. The inflammatory infiltrate was distributed predominantly around superficial dermal vessels and extended focally into the interstitium. Eosinophils were present in small but conspicuous numbers, supporting the possibility of a drug-induced pustular eruption. No significant vasculitic changes were identified. In addition, no evidence of leukocytoclastic vasculitis, fibrinoid vessel wall necrosis, or significant erythrocyte extravasation was observed. Histopathological examination revealed superficial neutrophilic pustules, papillary dermal edema, and a mixed inflammatory infiltrate containing eosinophils. Although neutrophilic pustule formation may also occur in pustular psoriasis, the presence of eosinophils, absence of regular psoriasiform epidermal hyperplasia and rete ridge elongation, and the close temporal relationship with hydroxychloroquine exposure favored a diagnosis of drug-induced AGEP rather than generalized pustular psoriasis (Figure 2).

Clinically, the eruption was characterized predominantly by widespread erythematous and urticarial plaques with annular and targetoid morphology, accompanied by progressive confluence and marked superficial desquamation. Although discrete pustules were not a prominent feature on examination, histopathology subsequently demonstrated subcorneal sterile pustules, supporting the diagnosis of AGEP. No persistent thick psoriatic plaques or chronic psoriasiform skin changes were observed during the course of the disease.

Taken together, the clinicopathological findings were considered highly suggestive of AGEP and were subsequently supported by a EuroSCAR score of 7 (Table 2).

Although the EuroSCAR score classified the case as probable rather than definite AGEP, the diagnosis was strongly supported by the characteristic clinical presentation, compatible histopathological findings, leukocytosis, and recurrence of a similar eruption following re-exposure to hydroxychloroquine.

Microbiological evaluation, including appropriate cultures and laboratory testing, did not indicate the presence of an underlying infectious etiology, further supporting a drug-induced reaction.

Assessment of causality using the Naranjo Adverse Drug Reaction Probability Scale indicated a probable association between exposure to Hydroxychloroquine and the observed cutaneous reaction, with a score of 8.

During hospitalization, the patient was treated with systemic corticosteroids, antihistamines, and supportive measures, resulting in gradual clinical improvement. Hydroxychloroquine was discontinued, and strict avoidance was recommended.

After discharge, the patient continued regular dermatological follow-up. A gradual regression of lesions was observed, with residual erythema and scaling but without the appearance of new pustular lesions. Symptomatic treatment with emollients led to further improvement. A clinical pharmacologist concluded that the reaction was highly likely to be drug-induced, emphasizing the significance of the positive rechallenge, given that similar cutaneous manifestations had also occurred after previous exposure to the same medication.

## 4. Discussion

Acute Generalized Exanthematous Pustulosis represents a rare but significant adverse drug reaction that most commonly occurs shortly after exposure to the causative agent. In the context of Hydroxychloroquine use, AGEP is considered an uncommon but clinically relevant entity, with an increasing number of reported cases in the literature [23].

Our case demonstrates several features consistent with previous reports. First, the time interval between drug administration and symptom onset may be prolonged compared with other medications, often ranging between 2 and 3 weeks. Furthermore, the clinical presentation may be atypical and include urticarial lesions, annular plaques, and pustules, which may complicate the differential diagnosis with pustular psoriasis [4,16]. The prolonged clinical course and lack of complete resolution within 15 days resulted in a EuroSCAR classification of probable rather than definite AGEP. However, this pattern has been previously described in hydroxychloroquine-induced AGEP and is thought to be related to the drug’s prolonged elimination half-life.

Histopathologically, eosinophilic infiltrates, papillary dermal edema, and the absence of significant psoriasiform epidermal hyperplasia are considered useful features favoring AGEP over generalized pustular psoriasis [22]. In our case, the combination of subcorneal pustules, scattered eosinophils, mild spongiosis, and the absence of regular psoriasiform changes supported a drug-induced pustular eruption consistent with AGEP. Nevertheless, histopathological overlap between AGEP and pustular psoriasis remains a recognized diagnostic challenge, particularly in atypical hydroxychloroquine-induced cases. Previous studies suggested that hydroxychloroquine may unmask or induce pustular reactions in genetically predisposed individuals, potentially contributing to overlapping clinicopathological features [22]. Similar overlap with other severe cutaneous adverse reactions, including the Stevens–Johnson syndrome/toxic epidermal necrolysis (SJS/TEN) spectrum, has also been described in the literature, further complicating the diagnostic process [4,8]. Therefore, correlation between histopathological findings, clinical presentation, drug exposure history, and validated diagnostic scoring systems remains essential for accurate diagnosis.

The main differential diagnoses in this case included pustular psoriasis, drug reaction with eosinophilia and systemic symptoms (DRESS), and urticarial vasculitis. Pustular psoriasis was considered because of the disseminated pustular eruption; however, the absence of a prior history of psoriasis, the acute onset following hydroxychloroquine exposure, and the presence of eosinophil inflammatory infiltrates favored AGEP. DRESS syndrome was considered unlikely due to the absence of significant systemic involvement, lack of marked organ dysfunction, and only mildly elevated inflammatory markers despite eosinophilia [24]. Urticarial vasculitis was excluded based on the absence of vasculitic changes on histopathological examination and the transient nature of the urticarial lesions [25].

An additional diagnostic challenge in the present case is the recognized overlap between hydroxychloroquine-induced AGEP and hydroxychloroquine-triggered pustular psoriasis. Several authors have suggested that some cases initially diagnosed as AGEP may actually represent drug-induced pustular psoriasis occurring in genetically predisposed individuals [8,10,14]. This distinction may be particularly difficult in patients with prolonged disease duration, atypical clinical morphology, recurrent episodes, or overlapping histopathological features [8,14,22]. In our patient, the presence of annular erythematous plaques, urticarial lesions, and a relatively prolonged clinical course could be considered features compatible with either entity. However, the acute temporal relationship with hydroxychloroquine exposure, eosinophil-containing inflammatory infiltrates, papillary dermal edema, lack of significant psoriasiform epidermal hyperplasia, and the positive rechallenge provided additional supportive evidence for AGEP as the most likely diagnosis [24].

An important aspect of this case is the positive rechallenge, which provides supportive evidence for a causal relationship between hydroxychloroquine and the adverse reaction when interpreted together with the clinical and histopathological findings. Similar cases have also been confirmed through patch testing or accidental re-exposure, further emphasizing the immunological basis of this reaction [9]. Still, intentional re-exposure to the drug is generally not recommended for safety reasons; such cases, when documented, provide valuable supportive evidence for drug causality [26]. Although additional diagnostic methods, such as patch testing or the lymphocyte transformation test (LTT), may further confirm drug causality, they were not performed in this case.

The interpretation of the positive rechallenge should, however, be considered in light of the limited documentation available from the initial episode in 2017. Histopathological findings from that event were reported as nonspecific, and a definitive diagnosis was not established. Therefore, although the recurrence of a clinically similar eruption after re-exposure to hydroxychloroquine following a comparable latency period supports a causal relationship, it cannot be conclusively confirmed that both episodes represented the identical clinicopathological entity.

Recent studies have suggested that mutations in IL36RN and CARD14 may contribute to susceptibility to pustular reactions [10]. Because hydroxychloroquine may trigger pustular psoriasis in genetically predisposed individuals, the absence of IL36RN and CARD14 testing represents a limitation of the present report. Consequently, although the overall clinical presentation, histopathological findings, and drug causality assessment favored AGEP, an underlying genetic predisposition to pustular psoriasis cannot be completely excluded.

The primary treatment of AGEP consists of prompt discontinuation of the offending drug, which usually results in clinical improvement. Systemic corticosteroids may be considered in more severe or prolonged cases, while biologic therapy has been reported only in selected refractory patients [4,27].

Our case further emphasizes the importance of obtaining a detailed medical history, including previous drug reactions, as well as exercising caution when reintroducing potentially high-risk therapies, particularly in the setting of atypical presentations that may mimic other severe dermatoses. Early recognition and appropriate management are essential for preventing complications and achieving a favorable outcome.

Although positive rechallenge has been reported previously in hydroxychloroquine-induced AGEP, the present case adds to the existing literature by demonstrating an unusual combination of generalized pustular, annular, and urticarial lesions together with histopathological findings supporting AGEP. Furthermore, the case highlights the diagnostic challenges posed by the overlap between AGEP and hydroxychloroquine-triggered pustular psoriasis, particularly in the setting of recurrent episodes and prolonged disease duration. The detailed clinicopathological correlation and discussion of alternative diagnostic possibilities represent important contributions of the present report.

## 5. Conclusions

The presented case demonstrates that hydroxychloroquine may induce rare but clinically significant cutaneous reactions, including an overlap of urticarial and pustular lesions consistent with probable AGEP. A positive rechallenge provided supportive evidence for a causal relationship between hydroxychloroquine and the adverse reaction. Although probable AGEP was considered the most likely diagnosis based on the overall clinical, histopathological, and causality assessment findings, overlap with hydroxychloroquine-triggered pustular psoriasis cannot be completely excluded, particularly in the absence of genetic testing. Prompt recognition and discontinuation of the drug are crucial for achieving a favorable outcome and preventing recurrence of the reaction. Accurate diagnosis relies on careful integration of the clinical presentation, drug exposure history, histopathological findings, and validated diagnostic scoring systems.

## Figures and Tables

**Table 1:** Published cases of hydroxychloroquine-induced AGEP compared with the present study.

Author, Year	Sex/Age	Underlying Disease	Latency After HCQ	Clinical Features	Histopathology	Rechallenge	Treatment/Outcome
Bailey et al., 2013 [15];	Female, 48 y	Dermatologic/rheumatologic disease	~2 weeks	Diffuse pustular eruption with fever	Compatible with AGEP	No	Resolution after withdrawal and steroids
Duman et al., 2017 [16];	Female, 42 y	Rheumatoid arthritis	~21 days	Generalized pruritic erythema, non-follicular pustules, targetoid lesions, fever, leukocytosis	Subcorneal pustules with neutrophilic infiltrate, acantholysis, spongiosis	Yes (negative patch test)	Steroids and dapsone; complete resolution
Mohaghegh et al., 2018 [7];	Female, 71 y	Rheumatoid factor-negative arthritis	14 days	Pustules, erythema, fever, leukocytosis; AGEP SJS overlap	Subcorneal pustules with neutrophilic infiltrate and dermal inflammation	No	HCQ stopped; IV steroids and IVIG; burn unit; recovery
Coleman et al., 2020 [17];	Female, 68 y	Cutaneous sarcoidosis	~4 weeks	Severe rash, skin sloughing, targetoid lesions, mucosal involvement; AGEP SJS overlap	Subcorneal pustules with neutrophilic infiltrate	No	Steroids and IVIG; prolonged taper; recovery
Hsieh et al., 2021 [20];	Female, 63 y	Suspected autoimmune disease	17 days	Scarlatiniform erythema, pustules, fever, neutrophilia	Subcorneal and intraepidermal pustules with neutrophilic infiltrate	No	HCQ stopped; corticosteroids; resolution
Kızıltepe et al., 2023 [18];	Female, 60 y	Sjögren’s syndrome	2–3 weeks	Persistent pustular eruption and erythema	Subcorneal pustules with neutrophilic infiltrate	No	Partial steroid response; methotrexate; resolution
Luo et al., 2023 [14];	Female (5 pts), mean 40.2 y	Rheumatic diseases	~12 days	Fever, generalized sterile pustules, leukocytosis	Compatible with AGEP	No	Withdrawal and corticosteroids; resolution
Zheng et al., 2024 [19];	Female	Autoimmune disease	Not specified	HCQ-induced AGEP	Compatible with AGEP	No	Resolution after withdrawal
Chen et al., 2023 [22];	Female, 53 y	Rheumatoid arthritis	3 days (re-exposure)	Recurrent pustular eruption (AGEP/PP overlap)	Subcorneal neutrophilic infiltrate	Yes	Improvement after discontinuation
Ali et al., 2026 [21];	Female, 53 y	Psoriatic arthritis	~2 weeks	Morbilliform eruption without visible pustules	Subcorneal pustules with neutrophils and eosinophils	No	Steroids; complete resolution
Present study	Female, 45 y	Rheumatoid arthritis	10 days	Annular erythematous plaques with sterile papulopustules and pruritus	Subcorneal pustules with neutrophils and eosinophils	Yes	Steroids; gradual recovery

**Table 2:** EuroSCAR Validation Score for Acute Generalized Exanthematous Pustulosis (AGEP) in the Present Case.

Criterion	Findings in the Present Case	Score
Pustules	Scattered non-follicular pustules within annular erythematous plaques; histopathology confirmed subcorneal pustulation	+2
Erythema	Generalized erythematous plaques involving the trunk and extremities	+2
Distribution	Rapidly progressive generalized eruption affecting the trunk and limbs	+2
Post-pustular desquamation	Desquamation and exfoliative scaling documented during lesion regression	+1
Mucosal involvement	No mucosal involvement reported	0
Onset	Eruption developed within 10 days of hydroxychloroquine exposure	0
Resolution	Complete resolution did not occur within 15 days	−4
Temperature ≥38 °C	Patient remained afebrile throughout hospitalization	0
Leukocyte count ≥7000/mm^3^	Leukocytosis documented (13.6 × 10^9^/L)	+1
Histopathology	Subcorneal pustules with neutrophilic infiltrates, mild spongiosis, and eosinophils; findings compatible with AGEP	+3
**Total EuroSCAR Score**		**7**

According to the EuroSCAR validation scoring system, scores are interpreted as follows: ≤0 = no AGEP; 1–4 = possible AGEP; 5–7 = probable AGEP; and 8–12 = definite AGEP.

## Data Availability

The original contributions presented in this study are included in the article. Further inquiries can be directed to the corresponding author.
